# Supplementary figures and images for: Identifying CD1c as a potential biomarker by the comprehensive exploration of tumor mutational burden and immune infiltration in diffuse large B cell lymphoma
Source: PeerJ. 2023 Dec 11;11:e16618. doi: 10.7717/peerj.16618 (PMC10720422; doi:10.7717/peerj.16618)

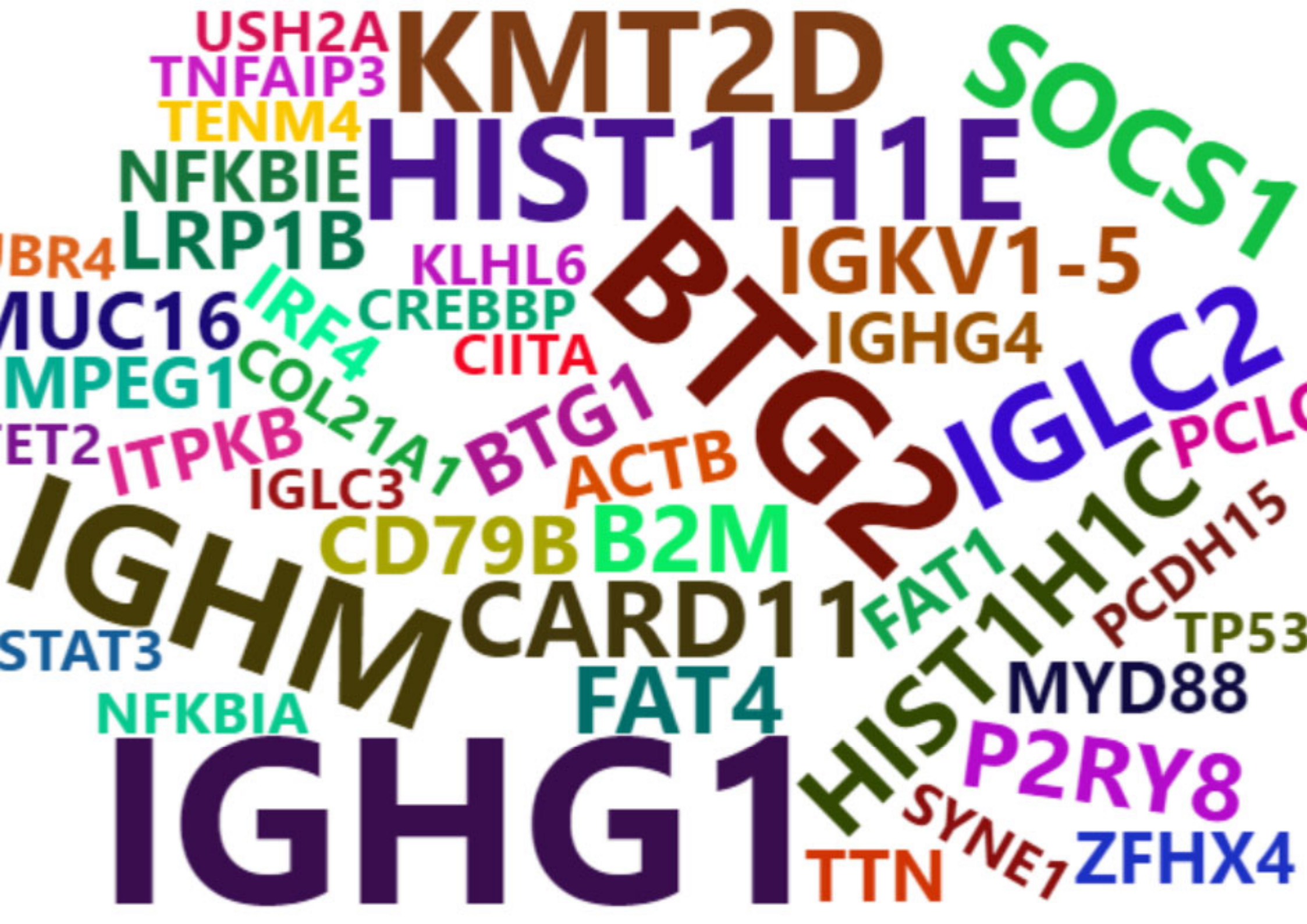

Supplement: Supplemental Information 1 [file peerj-11-16618-s001.pdf]

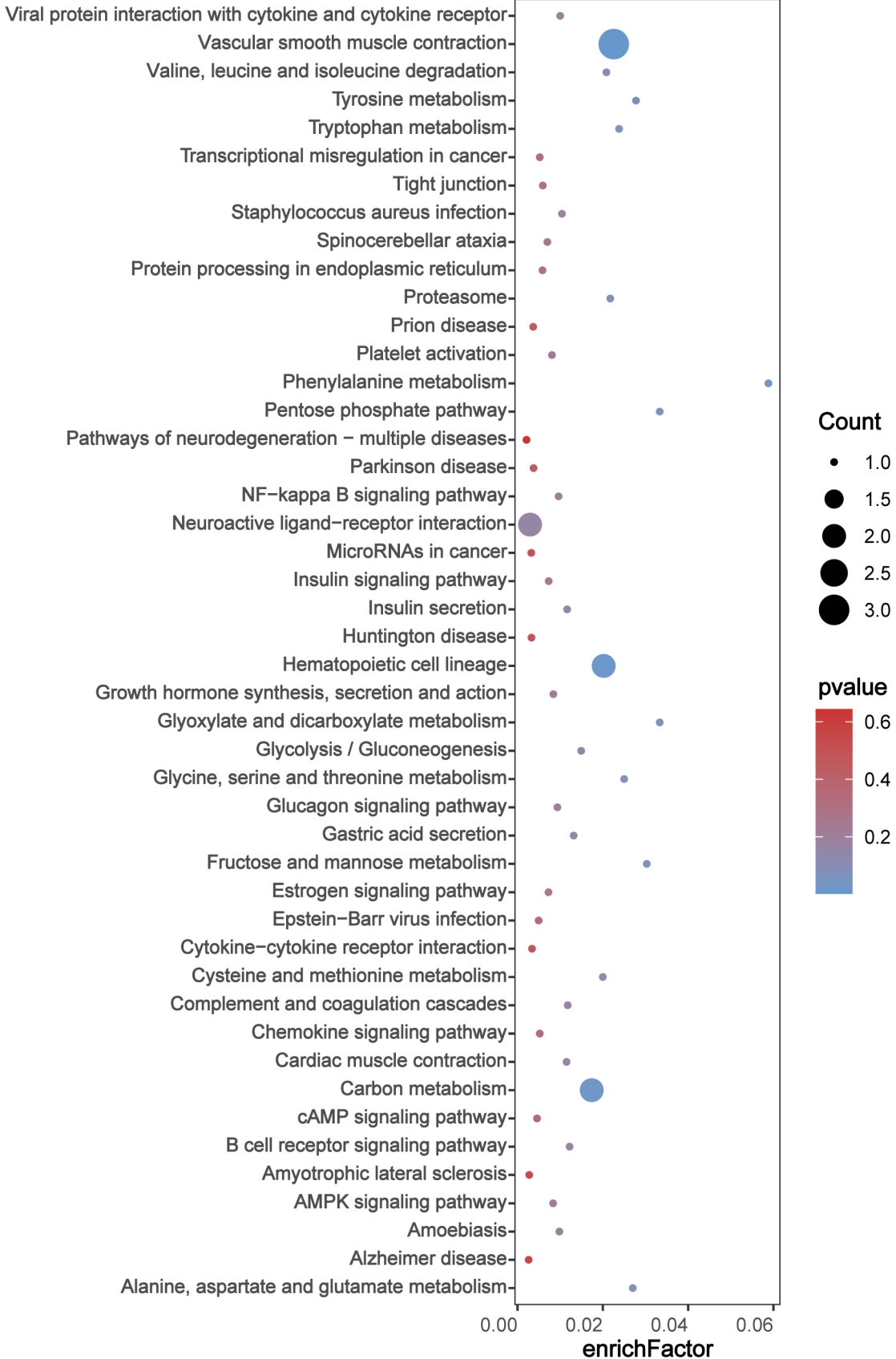

Supplement: Supplemental Information 2 [file peerj-11-16618-s002.pdf]

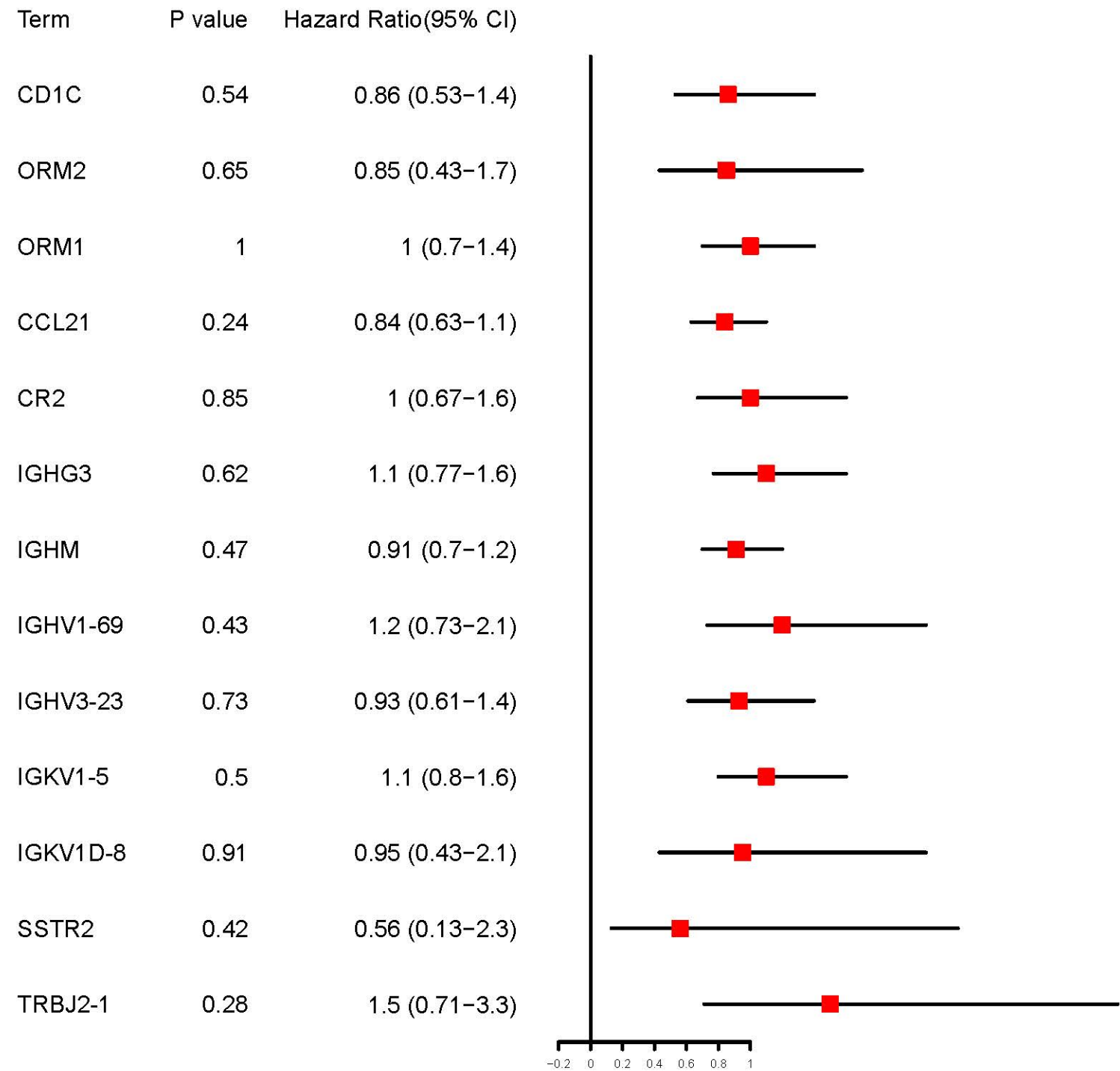

Supplement: Supplemental Information 3 [file peerj-11-16618-s003.pdf]
