# Supplementary material for: Identifying CD1c as a potential biomarker by the comprehensive exploration of tumor mutational burden and immune infiltration in diffuse large B cell lymphoma
Source: PeerJ. 2023 Dec 11;11:e16618. doi: 10.7717/peerj.16618 (PMC10720422; doi:10.7717/peerj.16618)
Supplement: Supplemental Information 4 — (A) GSE32918 (P < 0.0001). (B) GSE31312 (P = 0.034). (C) GSE10846 (P = 0.067). (D) GSE53786 (P = 0.041). (E) GSE87371 (P = 0.031). (F) GSE181063 (P =0.00011). [file peerj-11-16618-s004.pdf]

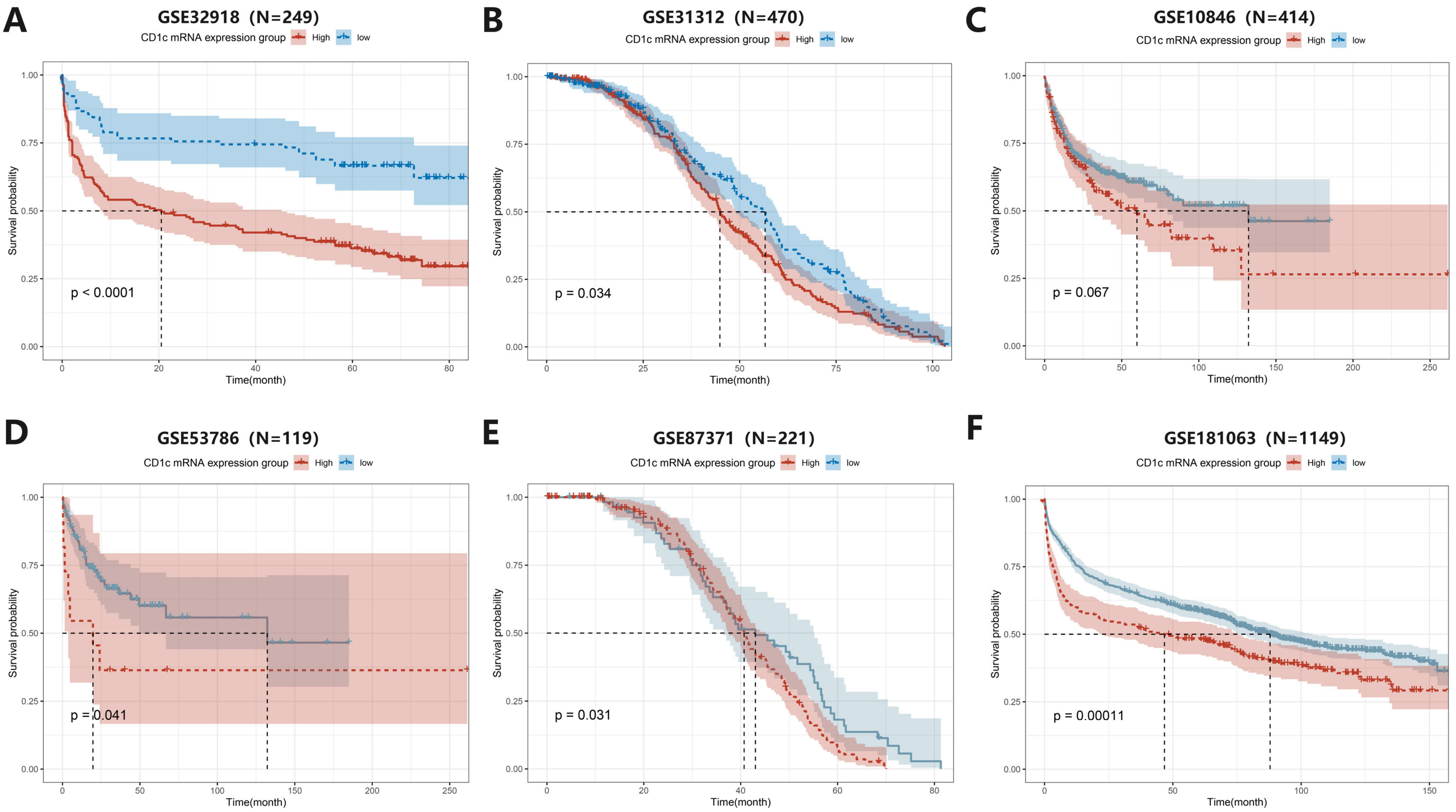
A
GSE32918 (N=249)
CD1c mRNA expression group High low
p < 0.0001
B
GSE31312 (N=470)
CD1c mRNA expression group High low
p = 0.034
C
GSE10846 (N=414)
CD1c mRNA expression group High low
p = 0.067
D
GSE53786 (N=119)
CD1c mRNA expression group High low
p = 0.041
E
GSE87371 (N=221)
CD1c mRNA expression group High low
p = 0.031
F
GSE181063 (N=1149)
CD1c mRNA expression group High low
p = 0.00011
Survival probability
Time(month)
