# Supplementary material for: Identifying CD1c as a potential biomarker by the comprehensive exploration of tumor mutational burden and immune infiltration in diffuse large B cell lymphoma
Source: PeerJ. 2023 Dec 11;11:e16618. doi: 10.7717/peerj.16618 (PMC10720422; doi:10.7717/peerj.16618)
Supplement: Supplemental Information 5 [file peerj-11-16618-s005.pdf]

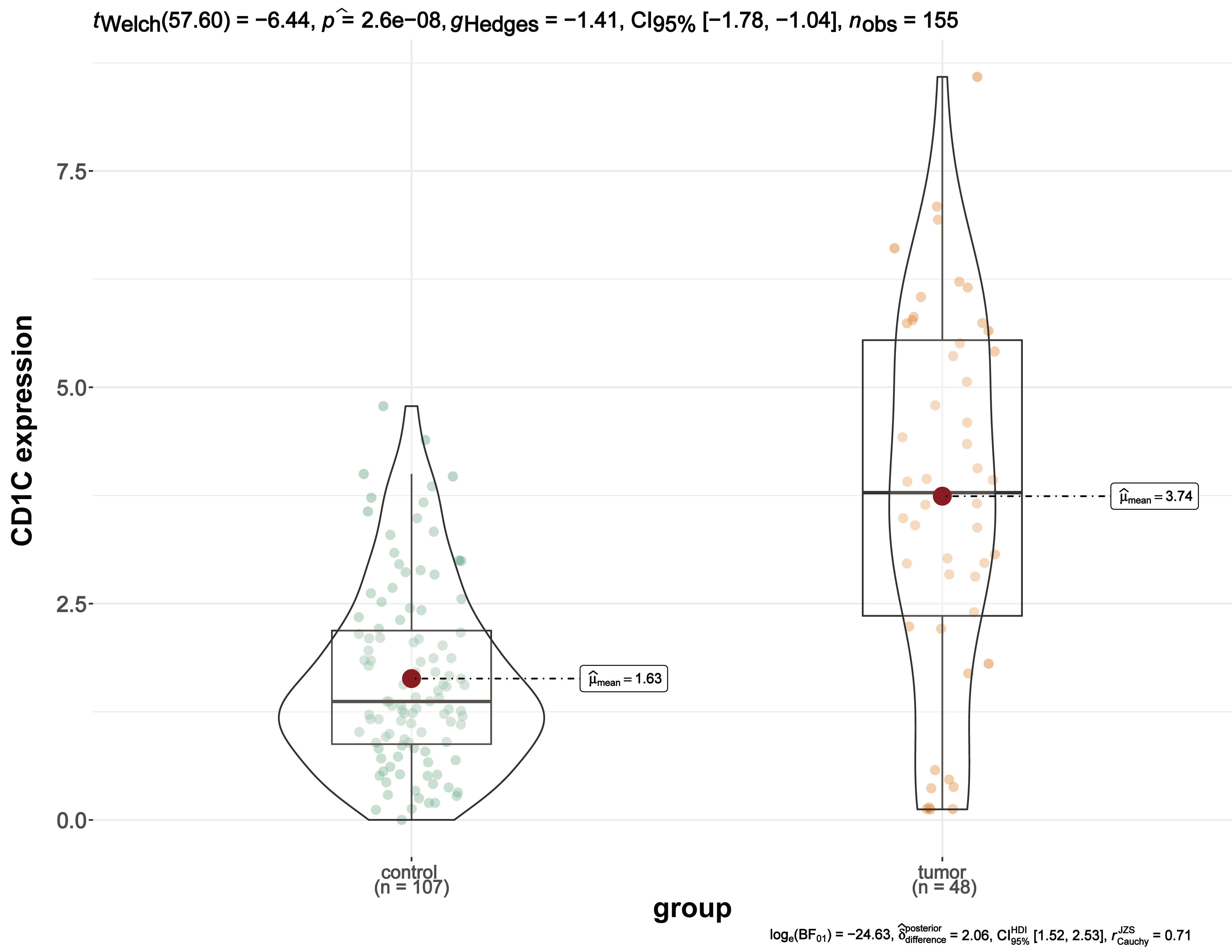

$t_{Welch}(57.60) = -6.44$, $\hat{p} = 2.6e-08$, $g_{Hedges} = -1.41$, $CI_{95\%}$ [−1.78, −1.04], $n_{obs} = 155$
CD1C expression
7.5
5.0
2.5
0.0
$\hat{\mu}_{mean} = 1.63$
$\hat{\mu}_{mean} = 3.74$
control
(n = 107)
tumor
(n = 48)
group
$\log_e(BF_{01}) = -24.63$, $\hat{\delta}^{posterior}_{difference} = 2.06$, $CI^{HDI}_{95\%}$ [1.52, 2.53], $r^{JZS}_{Cauchy} = 0.71$
