# Supplementary material for: Identifying CD1c as a potential biomarker by the comprehensive exploration of tumor mutational burden and immune infiltration in diffuse large B cell lymphoma
Source: PeerJ. 2023 Dec 11;11:e16618. doi: 10.7717/peerj.16618 (PMC10720422; doi:10.7717/peerj.16618)
Supplement: Supplemental Information 6 [file peerj-11-16618-s006.docx]

**Supplementary Table 1.** The differences in clinical characteristics between low- and high-TMB groups were obtained from the TCGA cohort.

| **TMB** | | | | | |
| --- | --- | --- | --- | --- | --- |
| **Age** | | **Total (N=36)** | **high (N=9)** | **low (N=27)** | **P-value** |
| < 65 | 25 (69.4%) | | 7 (77.8%) | 18 (66.7%) | 0.835 |
| ≥ 65 | 11 (30.6%) | | 2 (22.2%) | 9 (33.3%) |  |
| **B Symptoms** |  | |  |  |  |
| NO | 27 (75.0%) | | 8 (88.9%) | 19 (70.4%) | 0.191 |
| YES | 7 (19.4%) | | 0 (0%) | 7 (25.9%) |  |
| Unknown | 2 (5.6%) | | 1 (11.1%) | 1 (3.7%) |  |
| **Bone marrow involvement** | | | | | |
| NO | 23 (63.9%) | | 7 (77.8%) | 16 (59.3%) | 0.565 |
| YES | 1 (2.8%) | | 0 (0%) | 1 (3.7%) |  |
| Unknown | 12 (33.3%) | | 2 (22.2%) | 10 (37.0%) |  |
| **clinical_stage** |  | |  |  |  |
| Stage I | 6 (16.7%) | | 2 (22.2%) | 4 (14.8%) |  |
| Stage II | 15 (41.7%) | | 3 (33.3%) | 12 (44.4%) | 0.793 |
| Stage III | 2 (5.6%) | | 0 (0%) | 2 (7.4%) |  |
| Stage IV | 8 (22.2%) | | 2 (22.2%) | 6 (22.2%) |  |
| Unknown | 5 (13.9%) | | 2 (22.2%) | 3 (11.1%) |  |
| **EBV status** |  | |  |  |  |
| negative | 5 (13.9%) | | 3 (33.3%) | 2 (7.4%) |  |
| positive | 1 (2.8%) | | 0 (0%) | 1 (3.7%) | 0.135 |
| Unknown | 30 (83.3%) | | 6 (66.7%) | 24 (88.9%) |  |
| **Extranodal involvement** |  | |  |  |  |
| NO | 20 (55.6%) | | 6 (66.7%) | 14 (51.9%) |  |
| YES | 14 (38.9%) | | 2 (22.2%) | 12 (44.4%) | 0.409 |
| Unknown | 2 (5.6%) | | 1 (11.1%) | 1 (3.7%) |  |
| **IDH level** |  | |  |  |  |
| Negative | 5 (13.9%) | | 1 (11.1%) | 4 (14.8%) |  |
| Positive | 17 (47.2%) | | 4 (44.4%) | 13 (48.1%) | 0.913 |
| Unknown | 14 (38.9%) | | 4 (44.4%) | 10 (37.0%) |  |
| **Primary therapy outcome** |  | |  |  |  |
| Complete Remission/Response | 27 (75.0%) | | 6 (66.7%) | 21 (77.8%) |  |
| Partial Remission/Response | 2 (5.6%) | | 1 (11.1%) | 1 (3.7%) | 0.299 |
| Progressive Disease | 3 (8.3%) | | 2 (22.2%) | 1 (3.7%) |  |
| Stable Disease | 1 (2.8%) | | 0 (0%) | 1 (3.7%) |  |
| Unknown | 3 (8.3%) | | 0 (0%) | 3 (11.1%) |  |
| **Radiation therapy** |  | |  |  |  |
| NO | 29 (80.6%) | | 6 (66.7%) | 23 (85.2%) |  |
| YES | 5 (13.9%) | | 3 (33.3%) | 2 (7.4%) | 0.121 |
| Unknown | 2 (5.6%) | | 0 (0%) | 2 (7.4%) |  |
| **Gender** |  | |  |  |  |
| male | 15 (41.7%) | | 5 (55.6%) | 10 (37.0%) |  |
| female | 21 (58.3%) | | 4 (44.4%) | 17 (63.0%) | 0.558 |
| **Race** |  | |  |  |  |
| asian | 17 (47.2%) | | 5 (55.6%) | 12 (44.4%) | 0.847 |
| white | 19 (52.8%) | | 4 (44.4%) | 15 (55.6%) |  |
