# Supplementary material for: Identifying CD1c as a potential biomarker by the comprehensive exploration of tumor mutational burden and immune infiltration in diffuse large B cell lymphoma
Source: PeerJ. 2023 Dec 11;11:e16618. doi: 10.7717/peerj.16618 (PMC10720422; doi:10.7717/peerj.16618)
Supplement: Supplemental Information 7 [file peerj-11-16618-s007.docx]

**Supplementary Table 2.** Top GO items for differentially expressed genes.

| **Term** | **Description** | **Gene Ratio** | **p-value** | **q-value** | **Count** |
| --- | --- | --- | --- | --- | --- |
| GO:0002449 | lymphocyte mediated immunity | 9/42 | 8.94E-08 | 3.96E-05 | 9 |
| GO:0002460 | adaptive immune response based on somatic recombination of immune receptors built from immunoglobulin superfamily domains | 9/42 | 9.81E-08 | 3.96E-05 | 9 |
| GO:0016064 | immunoglobulin mediated immune response | 7/42 | 6.28E-07 | 0.000110844 | 7 |
| GO:0006958 | complement activation, classical pathway | 6/42 | 6.58E-07 | 0.000110844 | 6 |
| GO:0019724 | B cell mediated immunity | 7/42 | 6.86E-07 | 0.000110844 | 7 |
| GO:0002455 | humoral immune response mediated by circulating immunoglobulin | 6/42 | 1.15E-06 | 0.000154514 | 6 |
| GO:0006956 | complement activation | 6/42 | 2.57E-06 | 0.000296293 | 6 |
| GO:0030449 | regulation of complement activation | 5/42 | 5.48E-06 | 0.000553617 | 5 |
| GO:0002697 | regulation of immune effector process | 8/42 | 7.84E-06 | 0.000703996 | 8 |
| GO:0002920 | regulation of humoral immune response | 5/42 | 1.16E-05 | 0.000933956 | 5 |
