# Supplementary material for: Identifying CD1c as a potential biomarker by the comprehensive exploration of tumor mutational burden and immune infiltration in diffuse large B cell lymphoma
Source: PeerJ. 2023 Dec 11;11:e16618. doi: 10.7717/peerj.16618 (PMC10720422; doi:10.7717/peerj.16618)
Supplement: Supplemental Information 8 [file peerj-11-16618-s008.docx]

Supplementary Table 3. Top KEGG enrichment pathways for differentially expressed genes ordered by p-value (P < 0.05).

| **Term** | **Description** | **Gene Ratio** | **p-value** | **q-value** | **Gene ID** | **Count** |
| --- | --- | --- | --- | --- | --- | --- |
| hsa04270 | Vascular smooth muscle contraction | 3/17 | 0.002491 | 0.110125 | 72/5739/94274 | 3 |
| hsa04640 | Hematopoietic cell lineage | 2/17 | 0.017869 | 0.315996 | 1380/911 | 2 |
| hsa01200 | Carbon metabolism | 2/17 | 0.023676 | 0.315996 | 2731/8789 | 2 |
| hsa00360 | Phenylalanine metabolism | 1/17 | 0.035142 | 0.315996 | 259307 | 1 |
