# Supplementary material for: Identifying CD1c as a potential biomarker by the comprehensive exploration of tumor mutational burden and immune infiltration in diffuse large B cell lymphoma
Source: PeerJ. 2023 Dec 11;11:e16618. doi: 10.7717/peerj.16618 (PMC10720422; doi:10.7717/peerj.16618)
Supplement: Supplemental Information 9 [file peerj-11-16618-s009.docx]

**Supplementary Table 4. *CD1c* expression between normal tissue and diffuse large B cell lymphoma**.

| **Sample** | **Sample ID** | **Pathology ID** | **CD1c mRNA expression** | **CD1c protein expression** |
| --- | --- | --- | --- | --- |
| Sample01 | T1 | L2000519 | 1.00 | 0.55 |
| Sample02 | T2 | L2000214 | 0.27 | 8.73 |
| Sample03 | T3 | L2100150 | 0.69 | 2.08 |
| Sample04 | T4 | L2100157 | 1.20 | 14.55 |
| Sample05 | T5 | L2100202 | 48.38 | 36.17 |
| Sample06 | T6 | L2100204 | 2.51 | 2.08 |
| Sample07 | T7 | L2100213 | 10.62 | 0.00 |
| Sample08 | T8 | L2100246 | 33.38 | 1.37 |
| Sample09 | T9 | L2100254 | 1.07 | 0.20 |
| Sample10 | T10 | L2100271 | 0.83 | 0.03 |
| Sample11 | T11 | L2100287 | 7.10 | 0.49 |
| Sample12 | T12 | L2100408 | 0.66 | 0.52 |
| Sample13 | T13 | L2100295 | 17.86 | 5.96 |
| Sample14 | T14 | L2100417 | 7.65 | 0.42 |
| Sample15 | T15 | L2100415 | 4.95 | 17.49 |
| Sample16 | T16 | L2100454 | 13.00 | 2.34 |
| Sample17 | T17 | Z2139511 | 6.43 | NA |
| Sample18 | T18 | Z2152204 | 0.88 | NA |
| Sample19 | T19 | Z2152985 | 29.42 | NA |
| Sample20 | T20 | Z2157414 | 6.11 | NA |
| Sample21 | T21 | L1900028 | 60.27 | 12.28 |
| Sample22 | T22 | L1900160 | 8.84 | 1.42 |
| Sample23 | T23 | L1900164 | 67.77 | 5.33 |
| Sample24 | T24 | L1900191 | 64.93 | 0.50 |
| Sample25 | T25 | Z1919871 | 58.65 | 0.45 |
| Sample26 | T26 | Z1927492 | 56.01 | 0.49 |
| Sample27 | T27 | Z1959932 | 52.65 | 0.20 |
| Sample28 | T28 | Z1931327 | 37.04 | NA |
| Sample29 | T29 | Z1924788 | 54.24 | 7.34 |
| Sample30 | T30 | Z1918661 | 28.37 | NA |
| Sample31 | T31 | Z1905302 | 80.03 | NA |
| Sample32 | T32 | Z1911133 | 20.15 | 2.86 |
| Sample33 | T33 | Z1941053 | 29.69 | NA |
| Sample34 | T34 | Z1934519 | 36.00 | 4.85 |
| Sample35 | T35 | Z1933930 | 9.66 | NA |
| Sample36 | T36 | Z1900756 | 15.26 | NA |
| Sample37 | T37 | Z2008503 | 5.06 | NA |
| Sample38 | T38 | Z2013238 | 23.31 | 1.75 |
| Sample39 | T39 | Z2016528 | 2.94 | NA |
| Sample40 | T40 | Z2017865 | 33.60 | 0.85 |
| Sample41 | T41 | Z2017001 | 20.37 | 0.91 |
| Sample42 | T42 | Z2043475 | 13.37 | NA |
| Sample43 | T43 | Z2121652 | 9.24 | 9.11 |
| Sample44 | T44 | Z2114970 | 27.95 | NA |
| Sample45 | T45 | Z2122930 | NA | NA |
| Sample46 | T46 | Z2129658 | 14.92 | NA |
| Sample47 | T47 | Z2130933 | 8.95 | NA |
| Sample48 | T48 | Z2131460 | 20.21 | NA |
| Sample49 | T49 | Z2131475 | 19.18 | 1.82 |
| Sample50 | T50 | Z2131621 | 22.53 | 0.98 |
| Sample51 | T51 | Z2137167 | 41.08 | 1.04 |
| Sample52 | T52 | L1900193 | 52.92 | 5.88 |
| Sample53 | T53 | L1900272 | 44.57 | 0.82 |
| Sample54 | T54 | L2000538 | 20.35 | 13.47 |
| Sample55 | T55 | L2000580 | 19.69 | 14.77 |
| Sample56 | T56 | L2000381 | 8.48 | 2.31 |
| Sample57 | T57 | L2000293 | 15.43 | 1.63 |
| Sample58 | T58 | L2000076 | 6.23 | 4.91 |
| Sample59 | T59 | L2000023 | 20.09 | 0.92 |
| Sample60 | T60 | L1900498 | 26.92 | 1.64 |
| Sample61 | T61 | L1900460 | 8.73 | 12.66 |
| Sample62 | T62 | L1900202 | 62.11 | 3.07 |
| Sample63 | T63 | L1900008 | 26.14 | 2.72 |
| Sample64 | T64 | L1900135 | 36.21 | 7.29 |
| Sample65 | T65 | L1900138 | 36.56 | NA |
| Sample66 | T66 | Z2108388 | 55.25 | NA |
| Sample67 | T67 | Z2058451 | 67.55 | NA |
| Sample68 | T68 | Z2038723 | 88.75 | 0.14 |
| Sample69 | T69 | Z2054305 | 28.47 | NA |
| Sample70 | T70 | Z2017133 | 107.08 | NA |
| Sample71 | T71 | Z1953852 | 103.49 | NA |
| Sample72 | T72 | Z1900528 | 102.72 | 10.76 |
| Sample73 | T73 | Z1941882 | 90.22 | NA |
| Sample74 | T74 | Z2165832 | 78.68 | 3.30 |
| Sample75 | T75 | Z2161904 | 66.12 | 4.81 |
| Sample76 | T76 | Q2046427 | 134.10 | NA |
